# Pathogen exclusion from intestinal mucus and antimicrobial susceptibility of *Bifidobacterium* spp. strains from fecal donors

**DOI:** 10.20517/mrr.2024.43

**Published:** 2024-11-01

**Authors:** Aki Ronkainen, Imran Khan, Reetta Satokari

**Affiliations:** Human Microbiome Research Program, Faculty of Medicine, University of Helsinki, Helsinki 00290, Finland.

**Keywords:** Antagonism, antimicrobial resistance, bacteriotherapy, hydrolase, mucus degradation, next-generation probiotics

## Abstract

**Aim:** To study the ability of bifidobacterial strains isolated from fecal donors to prevent pathogens from adhering to intestinal mucus, along with their antimicrobial susceptibility.

**Methods:** Pathogen prevention was assessed through an *in vitro* adhesion assay using immobilized porcine mucus. Subsequently, bifidobacterial RNA-Seq data were analyzed to pinpoint glycoside hydrolases and glycosyltransferases possibly involved in mucus degradation affecting pathogen adhesion. The antimicrobial susceptibility of bifidobacterial strains was evaluated using *in vitro* susceptibility testing, followed by analysis of whole-genome sequencing data to reveal antimicrobial resistance genes.

**Results:** Bifidobacterial strains inhibited pathogen adhesion to intestinal mucus, with most strains reducing the adhesion levels of pathogens like *Escherichia coli*, *Listeria monocytogenes*, *Salmonella* Typhimurium, and *Staphylococcus aureus* by at least 70%. None of the strains significantly affected *Pseudomonas aeruginosa*, but they moderately reduced the adhesion of *Yersinia enterocolitica*. Gene expression analysis indicated that the more effective strains expressed higher levels of glycoside hydrolases, correlating with their pathogen exclusion capabilities. Antimicrobial susceptibility testing revealed that most strains were sensitive to several antibiotics, though some exhibited resistance to tobramycin, trimethoprim, and ciprofloxacin. Notably, one strain carried the *tetW* gene, conferring resistance to tetracycline.

**Conclusion:** The bifidobacterial strains characterized in this study show potential for bacteriotherapeutic applications due to their strong ability to interfere with the adhesion of pathogenic bacteria and their lack of alarming antimicrobial resistance patterns.

## INTRODUCTION

Fecal microbiota transplantation (FMT) is a medical procedure in which fecal material from a healthy donor is transferred to a recipient with dysbiotic gut microbiota^[1]^. FMT aims to restore the diversity and function of the microbiota, thereby alleviating a condition associated with dysbiosis^[1]^. While FMT has shown remarkable efficacy and safety in treating recurrent *Clostridioides difficile* (*C. difficile*) infection (rCDI)^[2-4]^, the procedure fundamentally differs from traditional probiotics use. Unlike probiotics, which involve the administration of specific, well-characterized strains, FMT introduces a complex and undefined microbial community, making it difficult to identify the exact species responsible for therapeutic effects^[1]^. This lack of precision poses potential risks, including the unintentional transfer of pathogens, microbes with antibiotic resistance genes, or microbial community that, in the long term, increases the risk of developing some diseases or disorders, underscoring the need for careful donor screening as well as safety and efficacy assessment^[5-7]^. On the other hand, the strength of FMT lies in its ability to introduce a diverse bacterial consortium, which is effective at correcting dysbiosis, as demonstrated in the treatment of rCDI^[2-4]^. More recently, complex mixtures of commensal bacteria have also shown effectiveness in preventing CDI recurrence^[8]^. In addition, FMT presents a powerful discovery platform for new bacterial strains with therapeutic potential, as it enables the identification, isolation, and characterization of gut commensals that successfully colonize the recipient. Bifidobacteria, typical commensals inhabiting the human gastrointestinal tract, have long been recognized for their beneficial effects on gut health, and thus present an excellent reservoir for novel strains with therapeutic potential^[9,10]^. However, any bacterial strain intended for any kind of probiotic use has to meet numerous selection criteria, such as sufficient characterization, desired efficacy, and safety^[11,12]^.

One desired characteristic of a probiotic strain is its adherence to intestinal mucus^[13]^. Among bifidobacteria, mucus adhesion is a strain-specific feature facilitated through different cellular components, such as pili and other proteins, and polysaccharides^[10]^. Besides promoting continuous residence in the gut, the adhesion of bifidobacteria may contribute to colonization resistance by limiting the adhesion of pathogens^[13,14]^. For instance, it was shown that a probiotic product containing bifidobacteria and lactobacilli was able to inhibit the colonization of a pathogenic *Escherichia coli* strain^[15]^. This effect relied on probiotic-induced enhanced mucin production by the Caco-2 cells, suggesting that the modulation of mucins by probiotics has a role in preventing pathogen infiltration in addition to plain competition for adhesion sites.

Apart from adherence and induction of mucus secretion, one aspect of pathogen exclusion is a probiotic strain’s ability to degrade intestinal mucus. Although degradation may be viewed as an undesired characteristic as it is associated with pathogenesis, degradative capabilities of commensals enable their persistence in the gut by providing means to obtain energy and carbon^[12,16,17]^. Moreover, commensals such as bifidobacteria have been shown to limit excess degradation by promoting further mucus production in the host cells^[18,19]^. The major structural components of mucus are mucins, heavily *O*- and *N*-glycosylated proteins with branched oligosaccharides^[13,18,19]^. Glycans are linked to serine and threonine residues in the protein, and comprise core structures composed of α- and β-linked *N*-acetyl-glucosamine, *N*-acetyl-galactosamine, and galactose. These core structures are further elongated and decorated with α-linked fucose, sialic acid, and sulfate residues. Bifidobacterial mucin degradation relies on a concerted action by glycosyl hydrolases^[18,19]^. The degradation involves action by fucosidases, sialidases, and sulfatases that first trim the glycans from their respective substrates, thereby enabling further core degradation by other glycosyl hydrolases. However, some bifidobacterial strains have been shown to degrade *N*-glycans even without previous trimming^[20]^. Indeed, genomic analyses have shown that the extent of degradative capabilities among *Bifidobacterium* species is a strain-specific feature^[17]^.

As antimicrobial resistance continues to be a global health concern, assessing resistance in new probiotic strains is an important issue for their safety^[21,22]^. Evidence on resistance raises questions about the transmission of resistance genes within the gut microbial community. On the other hand, resistance genes that are not encoded in mobile genetic elements may be a beneficial feature when probiotic strains are co-administered with antimicrobials, potentially alleviating dysbiosis resulting from the drugs^[22]^. Bifidobacteria have been documented to show resistance to aminoglycosides, quinolones, polypeptides, and mupirocin^[22]^.

Earlier, we tracked the transfer of bifidobacteria from FMT donors to patients with recurrent *C. difficile* infection, and isolated donor strains that had persisted in the patients for even up to one year^[23]^. In the follow-up study, we showed that the strains differed in their adherence to intestinal mucus, with the strong adherence being linked to the expression of pilin genes^[24]^. Furthermore, one of the adherent strains was shown to be particularly effective in alleviating antimicrobial-induced dysbiosis in mice after oral administration^[24]^. As the strains have shown many promising features for probiotic use, we continued to explore their therapeutic potential and safety by addressing the strains’ ability to inhibit pathogen adhesion to intestinal mucus and their antimicrobial susceptibility, respectively.

## METHODS

### Bacterial strains and culture conditions

The bacterial strains used in the study are listed in Table 1. All the *Bifidobacterium* spp. strains were originally isolated from FMT donors during our previous study^[23]^. The donors were healthy adult volunteers who underwent screening to determine their eligibility as fecal donors for a clinical study. The donors were screened according to the protocol used at the time of donation, as described previously^[25,26]^. In short, the donors had normal body weight [body mass index (BMI) 18.5-24.9], did not have any gastrointestinal symptoms, and had not taken antibiotics for the past 6 months. They were negative for *C. difficile* by selective cultivation and toxin A/B test, for enteric bacterial pathogens by selective cultivation, and for ova and other intestinal parasites by microscopy. Additionally, tests for HBV, HCV, HIV-1, HIV-2, and *Treponema pallidum* from serum were negative. Further tests included total blood count, C-reactive protein, creatinine, and liver enzyme levels from blood, all of which were within the normal range^[26]^. This study received approval from the Ethics Committee of the Hospital District of Helsinki and Uusimaa, Finland (DnroHUS124/13/03/01/11), and the participants in the study provided informed consent^[23,25]^. The strains representing pathogens were obtained from American Type Culture Collection (ATCC, United States), German Collection of Microorganisms and Cell Cultures GmbH (DSMZ, Germany), or Vita Laboratories, Inc. (VITA, Finland). The pathogens labeled with VITA are established reference strains for clinical diagnostics at Vita Laboratories, Inc., Helsinki, Finland.

**Table 1:** Bacterial strains used in the study

**Bacterial strain**	
***Bifidobacterium* sp.^1^**	**Adhesion level^2^**
*Bifidobacterium adolescentis* DX_pv1	High (~12%)
*B. longum* DX_pv18	Low (~3%)
*B. longum* DX_pv23	High (~12%)
*B. longum* DX_pv32	Low (~3%)
*B. longum* DY_pv11	High (~12%)
*Bifidobacterium pseudocatenulatum* DX_pv5	Moderate (~6%)
**Pathogen**	**Characteristics**
*Escherichia coli* ATCC 25922	
*Escherichia coli* VITA ESBL	ESBL producer
*Klebsiella pneumoniae* subsp. *pneumoniae* ATCC 13883	
*Listeria monocytogenes* VITA	
*Proteus mirabilis* DSM 4479	
*Pseudomonas aeruginosa* ATCC 27853	
*Salmonella enterica* subsp. *enterica* serovar Typhimurium ATCC 14028	
*Staphylococcus aureus* subsp. *aureus* ATCC 25923	
*Yersinia enterocolitica* VITA	

1 Isolation and identification described in^[23]^. ^2^Adhesion levels described in^[24]^. *B. longum*: *Bifidobacterium longum.*

Depending on purpose, bifidobacteria were grown by using either Lactobacilli MRS Agar or Broth (Neogen Culture Media, CAT#NCM0035A/CAT#NCM0079A) supplemented with 0.5 g·L^-1^ of L-cysteine (Sigma-Aldrich, CAT#30129) (hereafter MRSc agar/broth) under anaerobic conditions at 37 °C for 48 ± 4 h. The pathogens were grown by using either Columbia Agar supplemented with 5% (v/v) of horse blood (Tammer BioLab, CAT#T253; hereafter CB agar) or Brain Heart Infusion Broth (Neogen Culture Media, CAT#NCM0016A) supplemented with 5% (w/v) of yeast extract (Neogen Culture Media, CAT#NCM0218A) (hereafter BHIS broth) under 5% CO_2_ atmosphere at 37 °C for 18 ± 2 h.

### Antagonistic adhesion assay

Bifidobacterial strains used in the study differ in their ability to adhere to intestinal mucus [Table 1]. Here, the strains were subjected to an antagonistic adhesion assay to explore whether they could exclude selected pathogens [Table 1] from adhering to mucus. Each bifidobacterial strain was prepared for the assay by suspending a single colony from MRSc agar into 500 μL of 1X phosphate-buffered saline (PBS). After mixing, two aliquots of 200 μL were pipetted in parallel into MRSc broth supplemented with and without 10 μL·mL^-1^ of tritiated thymidine (17,6 Ci mmol^-1^, PerkinElmer, CAT#NET355005MC) with the resulting cultures grown as described above. Pathogens were prepared in a similar fashion, except that colonies were picked from CB agar and suspended only in thymidine-supplemented BHIS broth. The bacterial cultures labeled with thymidine were utilized to determine individual adhesion levels. Unlabeled bifidobacterial cultures were utilized to treat the mucus prior to the introduction of labeled pathogens, enabling the evaluation of the treatment’s impact on individual adhesion levels. The assay was performed following previously described methods^[24,27]^. Mucin from the porcine stomach (Sigma-Aldrich, CAT#M2378) was immobilized to MaxiSorp™ microtiter plate (Thermo Scientific, CAT#445101) by overnight incubation at 4 °C. The resulting mucus plate containing 75 ng of mucin per well was washed three times with 200 μL of PBS. The bacterial cultures prepared for the assay were centrifuged to separate the medium from the cells that were then washed with, dissolved in, and adjusted with PBS to reach the optical density (OD_600_) of 0.25.

The individual adhesion levels were determined as follows: For each suspension, three replicates of 100 μL were pipetted on the mucus plate that was then incubated at 37 °C for 1 h. Each suspension was also used for another three replicates of 100 μL that were pipetted into OptiPhase HiSafe™ 3 liquid scintillation cocktail (PerkinElmer, CAT#1200.437) to serve as a reference for the cells pipetted on the mucus plate. After incubation, the wells of the mucus plate were washed three times with PBS and treated with 100 μL of 1% SDS - 0.1 M NaOH at 37 °C overnight. The contents of each well were transferred into OptiPhase HiSafe™ 3 and measured with the Wallac Winspectral 1414 liquid scintillation counter (PerkinElmer) along with the references. For each strain, adhesion level (%) was calculated from the ratio between the radioactivity recorded from the adhered cells and that of the pipetted cells using the arithmetic means of technical replicates ± standard deviation.

The evaluation of bifidobacterial strains’ impact on the adhesion of pathogens was conducted in parallel with the determination of individual adhesion levels. The assay was performed on a separate uninoculated mucus plate by using the same H^3^-thymidine-labeled suspensions of the pathogens and the unlabeled suspensions of the bifidobacterial strains. Here, the wells of the mucus plate were pretreated with unlabeled bifidobacterial strains (100 μL of suspension per well, three replicates per strain) by incubation at 37 °C for 1 h followed by a washing step (three times with 200 μL of PBS). This was followed by pipetting the labeled pathogens on the plate and proceeding as described above. The bifidobacterial impact was evaluated by comparing a pathogen’s adhesion level (%) with or without the pretreatment with a bifidobacterial strain. All the experiments were done in three biological replicates.

### Analysis of bifidobacterial glycoside hydrolase and glycosyl hydrolase expression

In our previous study^[24]^, we conducted a differential gene expression analysis for the same *Bifidobacterium* spp. strains used in this study [Table 1] with the strains grown therein under identical conditions. In this study, we used the previously produced RNA-Seq data to explore the expression of genes that might take part in mucin degradation.

Briefly, the cells were collected during the adhesion assays from unlabeled cultures (see above) of three separate experiments, stored in RNAlater® (Thermo Fisher, CAT#AM7020), and subjected to chemical and mechanical lysis by using MetaPolyzyme (Sigma-Aldrich, CAT#MAC4L) and repeated bead-beating with Fastprep®-24 instrument (MP Biomedicals), respectively. Total RNA was extracted from the homogenates with RNeasy Minikit (Qiagen, CAT#74104), including the DNase I treatment. The samples were sent for RNA-Seq to the Biomedicum Functional Genomics Unit at the University of Helsinki (Finland), wherein the ribo-depleted RNA was used for the preparation of indexed library that was sequenced on NextSeq 500 using NextSeq High Output 75 cycle flow cell (Illumina).

The raw FASTQ files were checked and controlled for quality with fastp, FastQC and MultiQC^[28-30]^. The RNA-Seq data were deposited to the NCBI SRA database under the accession number PRJNA930167^[24]^. The genomes of all six bifidobacterial strains were retrieved from the European Nucleotide Archive, and the four *Bifidobacterium longum* (*B. longum*) strains were employed to create pangenome with Roary^[23,31]^. The reads were aligned to the corresponding reference genome with the read counts used for the estimation of differentially expressed genes (DEGs) with edgeR^[32-34]^. The pairwise transcriptome comparisons of DEGs were conducted using an adjusted *P*-value threshold of < 0.5 and an absolute log2 (fold change) value of > 1. Expressed genes were screened for glycoside hydrolases and glycosyl transferases belonging to the enzyme classes 3.2 and 2.4, respectively.

### Antimicrobial susceptibility testing and resistance analysis

Bifidobacterial strains were subjected to antimicrobial susceptibility testing by employing the disc diffusion method. The testing was performed by picking colonies from MRSc agar into 500 μL of 0.9% NaCl to yield a suspension corresponding to the turbidity of McFarland standard 0.5. An aliquot of suspension was spread on MRSc agar with antimicrobial discs (Oxoid, Thermo Fisher Scientific) placed on the agar plate. The array of discs included amoxicillin/clavulanic acid 30 μg (CAT#CT0223B), ampicillin 2 μg (CAT#CT0002B), ceftazidime 10 μg (CAT#CT1629B), ceftriaxone 30 μg (CAT#CT0417B), cefuroxime sodium 30 μg (CAT#CT0127B), ciprofloxacin 5 μg (CAT#CT0425B), clindamycin 2 μg (CAT#CT0064B), erythromycin 15 μg (CAT#CT0020B), gentamicin 10 μg (CAT#CT0072B), imipenem 10 μg (CAT#CT0455B), levofloxacin 5 μg (CAT#CT1587B), mecillinam 10 μg (CAT#CT0096B), meropenem 10 μg (CAT#CT0774B), metronidazole 5 μg (CAT#CT0067B), nitrofurantoin 100 μg (CAT#CT0034B), penicillin G 1 unit (CAT#CT0152B), piperacillin/tazobactam 36 μg (CAT#CT1616B), rifampicin 5 μg (CAT#CT0207B), tetracycline 30 μg (CAT#CT0054B), tobramycin 10 μg (CAT#CT0056B), trimethoprim 5 μg (CAT#CT0076B), trimethoprim/sulfamethoxazole 1:19 25 μg (CAT#CT0052B), and vancomycin 5 μg (CAT#CT0188B). The plates were incubated under anaerobic conditions at 37 °C for 48 ± 4 h. Strains’ susceptibility for a given antimicrobial was assessed by measuring the inhibition zone induced by the disc. The disc diffusion method indicated some antimicrobial resistance among the bifidobacterial strains. To detect the underlying genetic resistance mechanisms, the genomes of the strains were searched for resistance genes using ResFinder^[35]^.

### Statistical analysis

For adhesion assays, data normality distribution was tested using the Shapiro–Wilk test. For bifidobacteria, the statistical significance of the data was evaluated by one-way analysis of variance (ANOVA) with Tukey multiple comparisons test. For other bacteria, one-way ANOVA with Dunnett multiple comparisons test was used. The tests were carried out in bioRender.com employing R (version 4.2.2). Statistical analysis of sequence data is described in the respective method section.

## RESULTS

### Bifidobacteria interfere with pathogen adhesion to intestinal mucus

**Figure 1:**
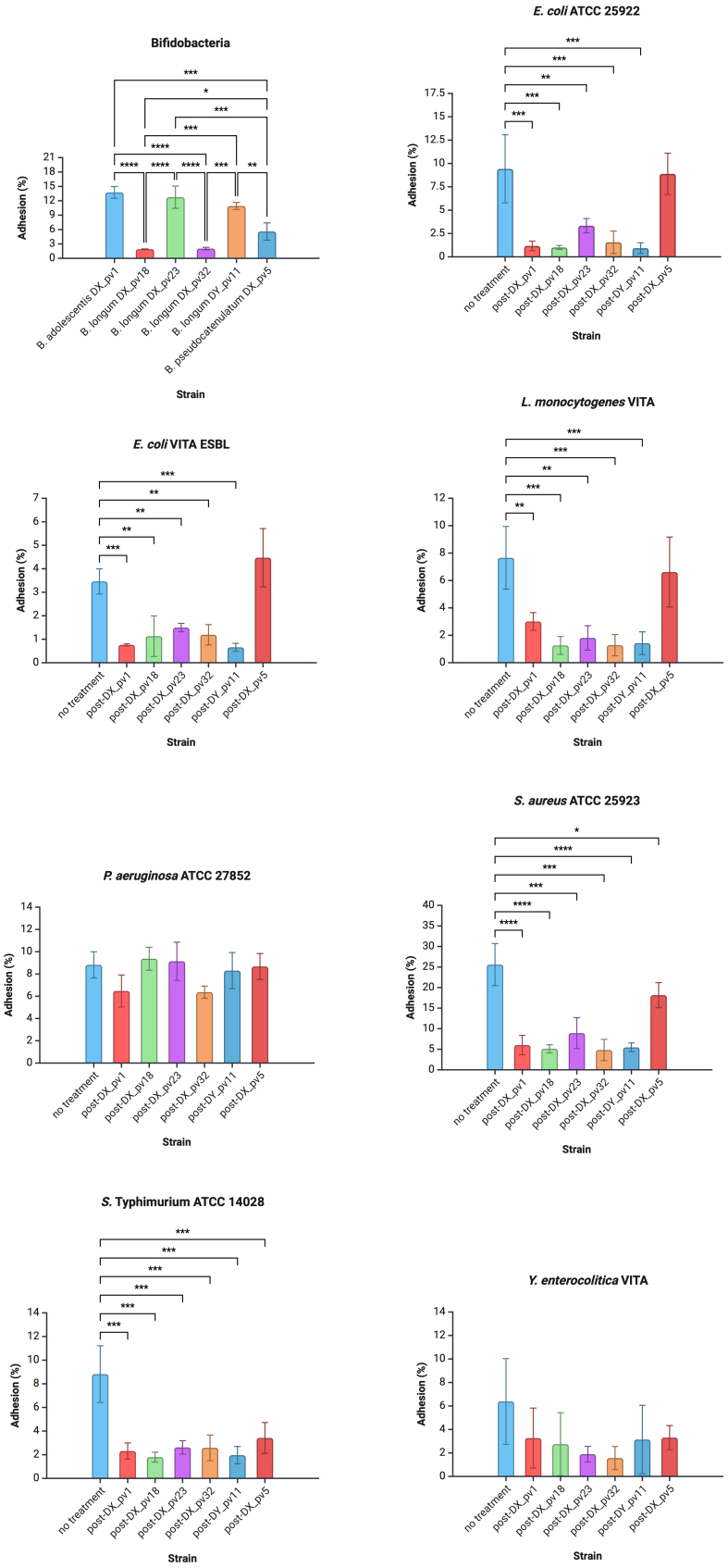
Adhesion of *Bifidobacterium* spp. and pathogens to intestinal mucus and the effect of bifidobacteria on the adhesion of pathogens. In the first panel, individual adhesion levels (%) of bifidobacterial strains are shown. In the other panels, the “no treatment” condition represents the adhesion level of the pathogen on intact mucus, followed by the adhesion level after the mucus has been pretreated with a given bifidobacterial strain. Data are presented as mean adhesion percentages calculated from three independent experiments, with error bars indicating standard deviation. Data normality distribution was tested using Shapiro–Wilk test. For bifidobacteria, the statistical significance of the data was evaluated by one-way ANOVA with Tukey multiple comparisons test. For other bacteria, one-way ANOVA with Dunnett multiple comparisons test was used. ^*^P < 0.05, ^**^P < 0.01, ^***^P < 0.001, ^****^P < 0.0001. Created with BioRender.com. ANOVA: Analysis of variance.

Previously, we evaluated the capability of our *Bifidobacterium* spp. strains [Table 1] to adhere to intestinal mucus. Here, the same assay was repeated and carried out also for the pathogens [Figure 1]. The bifidobacterial strains showed similar adhesion patterns as earlier^[24]^: *B. longum* strains DX_pv18 and DX_pv32 showed poor adhesion (adhesion level in average < 2%), while *B. adolescentis* DX_pv1 and *B. longum* strains DX_pv23 and DY_pv11 showed high adherence (> 10%), and *B. pseudocatenulatum* DX_pv5 showed moderate adherence (4%-6%) [Figure 1]. The pathogens displayed different adhesion levels: *E. coli* ATCC 25922, *P. aeruginosa* ATCC 27853, *S. aureus* ATCC 25923, and *S.* Typhimurium ATCC 14028 were considered highly adherent (adhesion level in average > 8%), *E. coli* VITA ESBL, *L. monocytogenes* VITA, and *Y. enterocolitica* VITA moderately adherent (4%-6%) [Figure 1], and *K. pneumoniae* ATCC 13883 and *P. mirabilis* DSM 4479 poorly adherent (< 2%) (data not shown).

Next, we used antagonistic adhesion assay to explore whether the bifidobacterial strains were able to inhibit pathogens from adhering to intestinal mucus. *K. pneumoniae* ATCC 13883 and *P. mirabilis* DSM 4479 were excluded from the analysis due to their minimal or low adhesion levels, respectively (data not shown), which could result in inaccurate evaluation of their inhibition. The results of the inhibition for pathogen adhesion are presented in Figure 1, illustrating the adhesion percentages relative to their original adhesion level. Based on the results, *B. longum* strains DX_pv18, DX_pv32, and DY_pv11 reduced the adhesion levels of *E. coli* ATCC 25922, *E. coli* VITA ESBL, *L. monocytogenes* VITA, *S.* Typhimurium ATCC 14028, and *S. aureus* ATCC 25923 in a similar efficacy (residual adhesion < 30%). *B. adolescentis* DX_pv1 seems to have a similar effect, except being somewhat less effective in inhibiting *L. monocytogenes*. *B. longum* strain DX_pv23 also reduces the adhesion of aforementioned strains, albeit not as efficiently. *B. pseudocatenulatum* DX_pv5 does not seem to be very efficient in excluding pathogens but showed some efficacy in inhibiting *S. aureus* and *S.* Typhimurium. Interestingly, none of the bifidobacterial strains interferes effectively with the adhesion of *P. aeruginosa* ATCC 27853. All the bifidobacterial strains reduce the adhesion level of *Y. enterocolitica* VITA moderately, but there was a high variation in the results, which may reflect the inconsistent *in vitro* adhesion properties of this pathogen (see Discussion).

### *B. longum* strains differ in the expression of glycoside hydrolases and glycosyl transferases

The results from the antagonistic adhesion assay indicate that even bifidobacterial strains with poor adherence are capable of inhibiting pathogen adhesion to intestinal mucus. As the bifidobacterial strains exhibited adhesion patterns similar to those observed previously^[24]^, we employed earlier whole transcriptome data to explore whether the strains expressed genes that might explain the exclusion effect.

At first, we looked at DEGs among the *B. longum* strains by comparing the expression of the poorly adherent strains DX_pv18 and DX_pv32 with the adherent strains DX_pv23 and DY_pv11. From a total of 593 genes expressed by the adherent strains, there were 202 upregulated and 391 downregulated genes. The genes were screened for glycoside hydrolases and glycosyl transferases belonging to the enzyme classes 3.2 and 2.4, respectively. The adherent strains prominently differed from the poorly adherent strains in the expression of a glycoside hydrolase from the enzyme family 43, a glycoside hydrolase encoded by *toxA*, and a cellulase family glycosylhydrolase [Supplementary Table 1]. Conversely, the most noticeable expression among the poorly adherent strains was detected in a glycoside hydrolase and in two glycosyl transferases [Supplementary Table 1]. Overall, the adhesion level and differential gene expression between strongly and poorly adherent strains did not explain a strain’s capacity to exclude pathogens.

**Figure 2:**
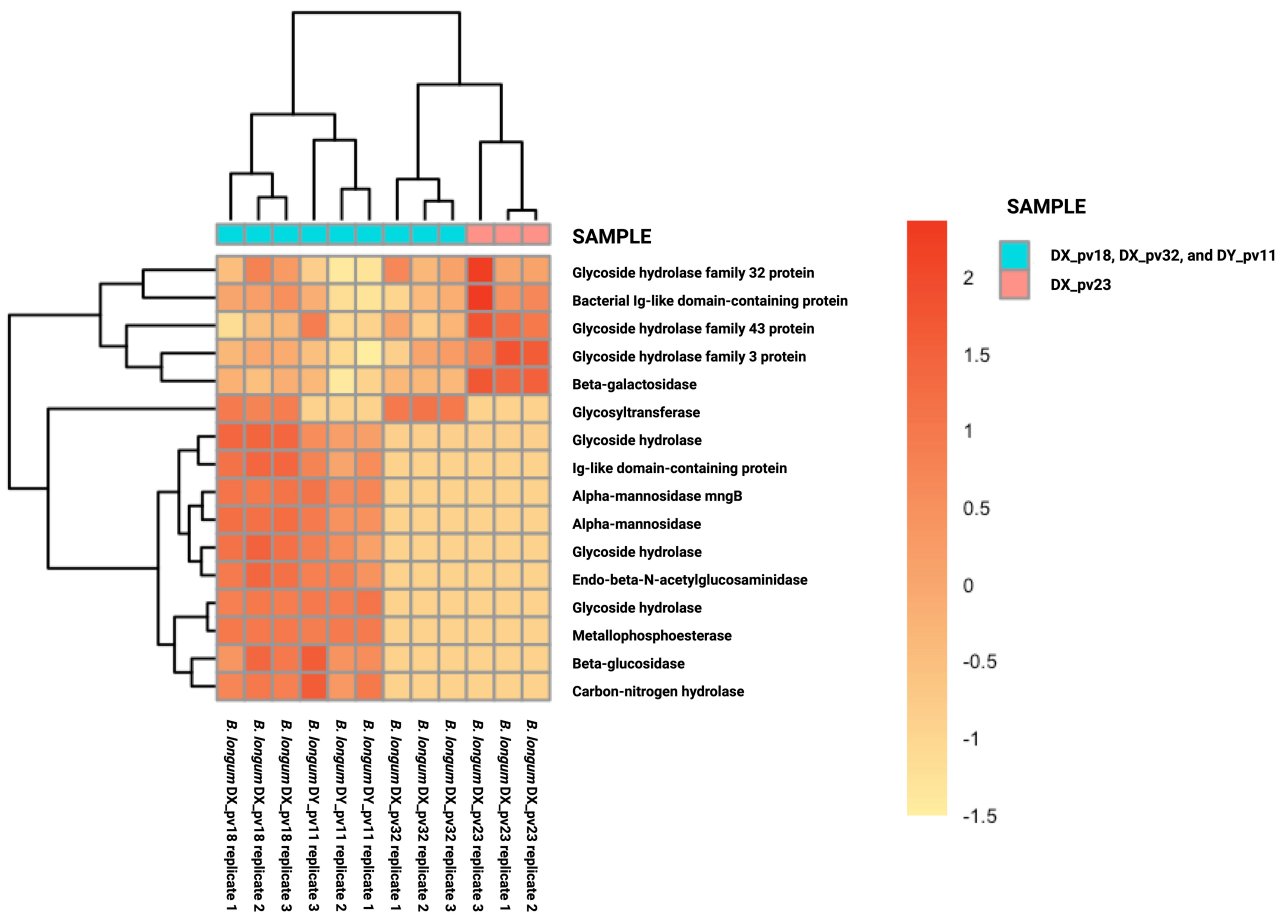
Differential gene expression among *B. longum* strains. Heatmap representing the upregulated and/or downregulated glycoside hydrolase and glycosyltransferase genes among *B. longum* strains. In the heat map, the expression of DX_18, DX_pv32, and DY_pv11 was compared to that of DX_pv23. *B. longum*: *Bifidobacterium longum.*

**Figure 3:**
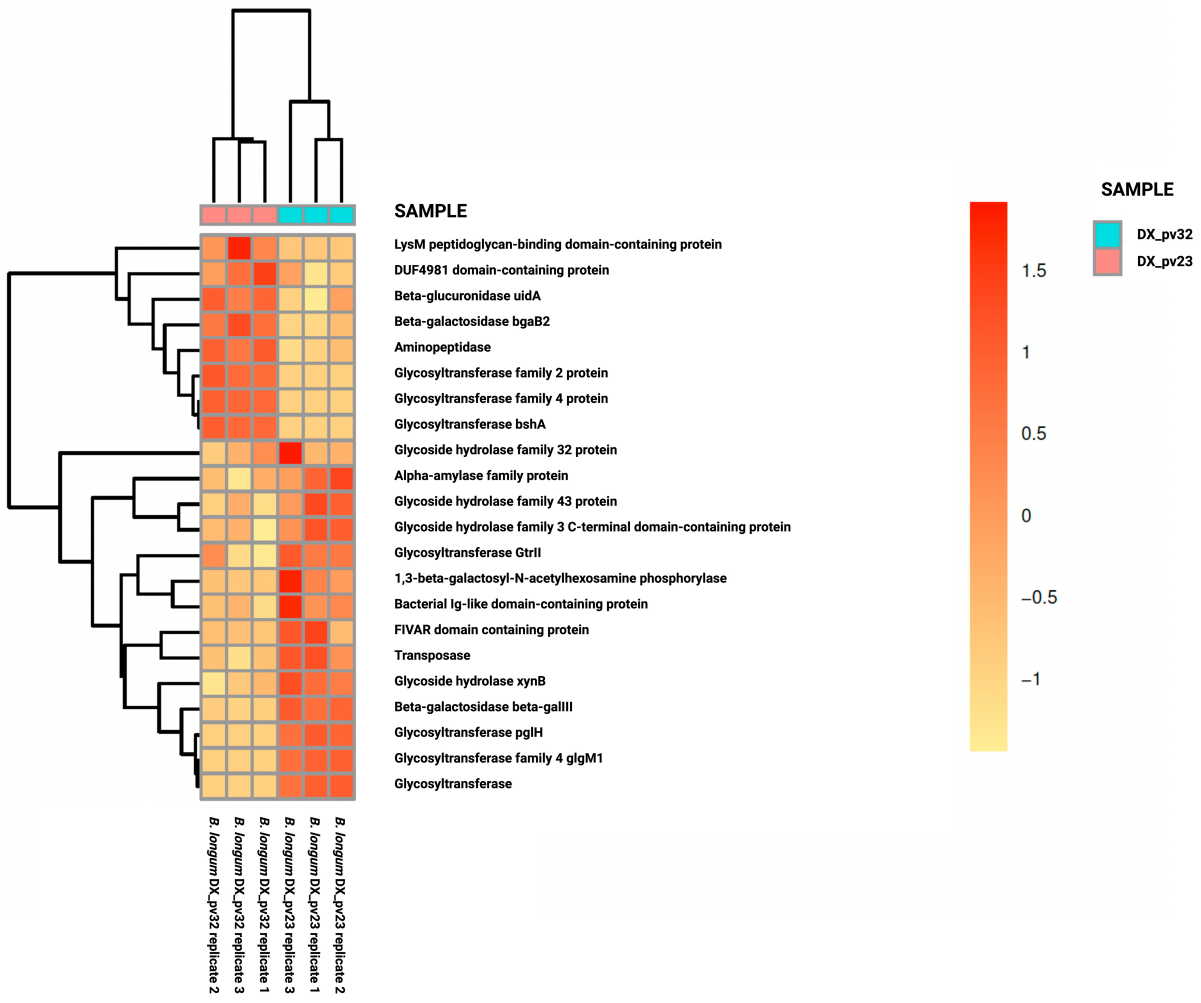
Differential gene expression between *B. longum* strains DX_pv32 and DX_pv23. Heatmap representing upregulated and/or downregulated glycoside hydrolase and glycosyl transferase genes. In the heat map, the expression of DX_pv32 was compared to that of DX_pv23. *B. longum*: *Bifidobacterium longum.*

Since the highly adherent DX_pv23 differed from the other *B. longum* strains by being less effective in inhibiting pathogen adhesion [Supplementary Table 1], we next compared its gene expression to the more effective *B. longum* strains DX_pv18, DX_pv32, and DY_pv11, i.e., we run differential gene expression comparison between the effective and less effective strains in inhibiting pathogen adhesion. From a total of 281 DEGs, there were 137 upregulated and 144 downregulated genes in DX_pv23. As for glycoside hydrolases and glycosyl transferases, the comparison did not reveal any high-level expression for these gene groups in DX_pv23 compared to the other strains [Figure 2, Supplementary Table 2]. Instead, the other strains showed expression of different glycosyl hydrolases such as alpha-mannosidase, beta-glucosidase, and endo-beta-*N*-acetylglucosaminidase, along with a glycosyl transferase [Figure 2], partly explaining their better performance in the inhibition of pathogen adhesion. Inspection of individual strains showed that while the DX_pv18 and DY_pv11 strains express glycoside hydrolases, including endo-beta-*N*-acetylglucosaminidase - an enzyme that hydrolyzes *N*-glycosylated proteins regardless of their prior trimming by other hydrolases, enabling straight-forward modification of mucus - DX_pv32 did not express the gene encoding these enzymes [Figure 2]. Followingly, the expression of DX_pv32 was compared to that of DX_pv23, which revealed that the strain DX_pv32 expresses various glycoside hydrolases at a higher level than DX_pv23, which may be related to its superior pathogen exclusion capacity [Figure 3]. Thus, the pathogen exclusion capacity of the *B. longum* strains DX_pv18, DX_pv32, and DY_pv11, of which only DY_pv11 is highly adherent, seems to be related to their expression of glycosyl hydrolases but not to their adhesion.

### Bifidobacterial strains show low resistance to common antimicrobials

Finally, we were interested in the antimicrobial susceptibility of our bifidobacterial strains (*B. adolescentis* DX_pv1, *B. longum* strains DX_pv18, DX_pv23, DX_pv32, and DY_pv11, and *B. pseudocatenulatum* DX_pv5) and tested this with the disc diffusion method for 23 antibiotics [Table 2]. As bifidobacteria are generally regarded as safe organisms, there are no established guidelines for interpreting clinical breakpoints, allowing for the use of specific cut-off values to define a strain as sensitive or resistant to a given antimicrobial. However, our data indicate that most drugs induce wide inhibition zones (> 20 mm), suggesting that the strains are sensitive to these drugs. Furthermore, there are a few antimicrobials that do not affect the growth of strains, suggesting underlying resistance. All the studied strains seem to be resistant to tobramycin, trimethoprim, and trimethoprim/sulphamethoxazole (no inhibition zone outside the disc). All the *B. longum* strains (DX_pv18, DX_pv23, DX_pv32, and DY_pv11) seemed to be also resistant to ciprofloxacin, gentamicin, levofloxacin, and mecillinam. *B. adolescentis* DX_pv1 also showed resistance to ciprofloxacin. Genomic analysis of strains showed only one resistance gene among the strains. *B. adolescentis* DX_pv1 carried the *tetW* gene, conferring resistance to tetracycline as indicated by *in vitro* disc diffusion method.

**Table 2:** Antimicrobial susceptibility of bifidobacterial strains

**Antimicrobial**	***B. Adolescentis* DX_pv1**	***B. Longum* DX_pv18**	***B. Longum* DX_pv23**	***B. Longum* DX_pv32**	***B. Longum* DY_pv11**	***B. pseudocatenulatum* DX_pv5**
Amoxicillin/clavulanate	48	46	40	40	45	45
Ampicillin	45	24	24	24	27	29
Ceftazidime	44	28	24	19	16	27
Ceftriaxone	50	36	34	31	32	41
Cefuroxime	64	42	40	38	46	47
Ciprofloxacin	6	6	6	6	6	22
Clindamycin	50	45	43	51	54	44
Erythromycin	62	42	40	46	44	37
Gentamicin	6	6	6	6	6	6
Imipenem	35	26	30	27	28	37
Levofloxacin	27	6	6	11	6	25
Mecillinam	6	6	6	6	6	17
Meropenem	62	38	30	32	33	34
Metronidazole	6	6	6	6	6	6
Nitrofurantoin	44	42	32	43	64	41
Penicillin G	43	24	24	21	22	22
Piperacillin/tazobactam	56	45	43	40	58	42
Rifampicin	37	35	34	37	45	28
Tetracycline	6	37	42	43	38	42
Tobramycin	6	6	6	6	6	6
Trimethoprim	6	6	6	6	6	6
Trimethoprim/sulfamethoxazole	6	6	6	6	6	6
Vancomycin	25	26	26	28	30	21

The numbers indicate diameters of inhibition zones (mm), with a minimum of 6 mm representing the disc diameter.

## DISCUSSION

With the advent of personalized medicine, there is a great interest in developing new targeted strategies to treat diseases and disorders related to gut microbiota imbalance (e.g., irritable bowel syndrome, inflammatory bowel disease, metabolic syndrome)^[2,5]^. Consequently, there is a demand for efficiently colonizing microbes that can contribute to restoring the microbial balance. Studies on FMT have shown rather contrasting results regarding colonization and favorable clinical outcomes: One study^[4]^ linked favorable clinical outcomes to increased colonization of donor strains, while another study^[3]^ attributed clinical success to recipient features and the compatibility between donors and recipients. In our earlier study^[8]^, we tracked the transfer of bifidobacteria from FMT donors to recipients and showed that donor strain colonization ranged from transient to long-term, with the recipients exhibiting distinct bifidobacterial donor strain profiles. While the impact of specific strains within the donor’s microbiota on clinical outcomes remains unclear, the findings highlight the disparity described above. In essence, a bacterial strain intended for therapeutic use must exhibit properties that promote its colonization in a new host, while the host itself and its resident microbiota must be receptive to the strain. In the follow-up study^[24]^, we demonstrated that donor strains capable of long-term colonization differ in their adherence to intestinal mucus, and associated strong adhesion to the expression of pilin genes. Here, our aim was to explore the therapeutic potential and safety of the same donor strains.

At first, we examined whether our bifidobacterial strains are capable of inhibiting pathogen adhesion to intestinal mucus by exclusion. *B. adolescentis* DX_pv1 and *B. longum* strains DX_pv18, DX_pv23, DX_pv32, and DY_pv11 were effective in excluding *E. coli* ATCC 25922, *E. coli* VITA ESBL, *L. monocytogenes* VITA, *S. aureus* ATCC 25923, and *S.* Typhimurium ATCC 14028 as the proportion of adhered cells decreased by roughly 60%-90% after pretreating the mucus layer with an individual bifidobacterial strain. Unlike the other bifidobacterial strains, *B. pseudocatenulatum* DX_pv5 did not show significant exclusion except for *S. aureus* ATCC 25923 and *S.* Typhimurium ATCC 14028. Interestingly, the exclusion results for *Y. enterocolitica* VITA varied significantly between biological replicates across all bifidobacterial strains. Unlike the other pathogens, *Y. enterocolitica* exhibited inconsistent behavior, even after multiple repetitions, which appears to be inherent to this specific strain. Previous research has shown that *Y. enterocolitica* uses diverse strategies for adhering to mucus and is particularly sensitive to environmental stimuli^[36]^. We suspect that this variability is due to fluctuations in *Y. enterocolitica* adhesin expression in response to unknown factors, despite maintaining consistent growth conditions throughout the experiments. This observation aligns with earlier findings reported for *Y. enterocolitica*^[36]^. Finally, none of the bifidobacterial strains were able to exclude *P. aeruginosa* ATCC 27853, a representative species notorious for its strong adherence to mucus^[37]^. Furthermore, *P. aeruginosa* is particularly well-known for its ability to form biofilms, which protect the species from environmental stressors. It is interesting to note the contrasting effects of different environments on *P. aeruginosa* biofilm formation. While BHI broth promotes the growth, adhesion, and biofilm formation of *P. aeruginosa* under *in vitro* conditions, studies have shown that mucins, which are the key components of mucus, actually disrupt these biofilms^[38-40]^. Mucins separate bacterial cells and promote a planktonic state, which inhibits biofilm integrity^[39,40]^. This effect is unique to mucins, as other viscous polymers fail to produce similar biofilm disruption, highlighting mucins’ crucial role in regulating microbial virulence and preventing infections^[39,40]^. It remains debatable whether *P. aeruginosa* formed protective biofilms under the experimental conditions of this study, given the previously observed contrasting effects between BHI broth and mucins.

Our results are in line with previous studies that demonstrate bifidobacterial exclusion as a strain-dependent phenomenon^[41-43]^. Unlike some bifidobacteria in the referred studies, none of our strains increased the adhesion of pathogens. The adhesion percentages observed in pathogen exclusion provide valuable insight into the efficacy of bifidobacterial strains in preventing pathogen colonization in the gut. Reduced adhesion of pathogens like *E. coli* and *L. monocytogenes* suggests that bifidobacteria effectively compete for binding sites on intestinal mucus, lowering the risk of infection and pathogen colonization. Bacterial adhesion is the critical initial step in the process of invasion and translocation, and the competitive exclusion of pathogens from epithelial surfaces helps lower the risk of infection^[44]^. Overall, competitive exclusion is biologically significant as it helps maintain gut health, supports immune function, and highlights bifidobacteria’s potential as therapeutic agents in maintaining and restoring microbial balance and providing colonization resistance against pathogens.

Among the studied *B. longum* strains, the poorly adherent DX_pv18 and DX_pv32 showed similar or greater exclusion efficacy than the adherent DY_pv11 and DX_pv23, respectively, suggesting that the exclusion effect does not rely solely on the occupation of adhesion sites. This led us to hypothesize that the inhibition might result from the expression of mucus-degrading hydrolases, which could potentially disrupt the adhesion sites employed by the pathogens. We explored gene expression data of our strains to detect the expression of glycoside hydrolases and glycosyl transferases, aiming to understand the good pathogen exclusion capacity of the poorly adherent *Bifidobacterium* strains. The initial expression comparison between the poorly adherent and adherent strains revealed differences in the expression of only few transferases and hydrolases between the poorly adherent strains DX_pv18 and DX_pv32 and the highly adherent strains DY_pv11 and DX_pv23. In contrast, when the comparison was done according to the strains’ exclusion efficacy, namely comparing DX_pv23 to the rest, we noticed that the strains DX_pv18, DX_pv32, and DY_pv11 with better exclusion capacity expressed a wide pattern of hydrolases. Intriguingly, one of the expressed genes by DX_pv18 and DY_pv11 encodes endo-β-*N*-acetylglucosaminidase. Previously, it has been shown that specific strains of *B. breve*, *B. infantis*, and *B. longum* were capable of hydrolyzing *N*-glycosylated mucins without prior trimming by using this hydrolase^[20]^. Furthermore, it was shown that the hydrolase gene is located in the same gene cluster with α-mannosidase genes^[20]^. In this study, we detected the expression of α-mannosidase genes along with the endo-β-*N*-acetylglucosaminidase. The expression of both hydrolase types suggests the degradation of *N*-glycosylated glycans, as mannose is only present in *N*-linked glycans^[13]^. The degradation of *N*-glycosylated glycans might also contribute to the inability of *B. longum* strains to exclude *P. aeruginosa*. It has been suggested that this pathogen relies on the enzymatic capabilities of other bacteria to obtain *N*-glycosylated amines that are required for its pathogenesis^[37]^. DX_pv32 did not express the enzymes but other hydrolases, which may partly explain its ability to inhibit pathogen adhesion.

Our assumptions on the mechanisms of pathogen exclusion by our strains are currently based on the gene expression data. The data are derived from RNA extracted from cultures growing under identical conditions as used for the experiments described herein and from three biological replicates and, thus, can be considered representative. We consider the hypothesis of glycosyl hydrolases well justified but acknowledge that our observations should be validated by further analyses, i.e., by examining the actual mucus degradation in the presence of bifidobacterial strains. Furthermore, it is possible that there are other factors disrupting pathogen adhesion, such as bifidobacterial metabolites (e.g., bacteriocins)^[22]^, or other effector molecules acting directly on pathogen adhesins. However, our approach may provide clues on pathogen exclusion mechanisms that are currently not well understood. If mucus degradation is involved in pathogen exclusion, moderate degradation might prove to be a desired characteristic for probiotic strains in addition to efficient adherence.

We also assessed the antimicrobial susceptibility of our strains (*B. adolescentis* DX_pv1, *B. longum* strains DX_pv18, DX_pv23, DX_pv32, and DY_pv11, and *B. pseudocatenulatum* DX_pv5) to examine whether they harbor resistance that could question their potential use in bacteriotherapy. Bifidobacteria are usually sensitive to β-lactams (amoxycillin, ampicillin, cefalosporins, oxacillin, penicillin G, piperacillin), carbapenems (imipenem, meropenem), lincosamides (clindamycin), and macrolides (erythromycin)^[45]^, and all our strains were sensitive to these antimicrobials. Conversely, bifidobacteria have shown resistance to aminoglycosides (gentamicin, tobramycin), quinolones (ciprofloxacin, levofloxacin), and metronidazole^[45]^, of which our strains showed resistance to tobramycin (all strains), ciprofloxacin (all strains except *B. pseudocatenulatum* DX_pv5), levofloxacin (all *B. longum* strains). In addition, all our strains were sensitive to nitrofurantoin, rifampicin, and vancomycin, while some showed resistance to trimethoprim and trimethoprim/sulphamethoxazole (all strains) and mecillinam (all *B. longum* strains), which is in line with previous observations for these species^[45,46]^. Next, we analyzed WGS data to investigate the genetic basis for the observed resistance. Resistance in bifidobacteria is often intrinsic, arising from mechanisms like the absence of cytochrome-mediated drug transport or the presence of atypical enzymes, such as isoleucyl-tRNA synthetase^[47]^. These mechanisms typically pose a low risk of horizontal gene transfer, reducing concerns about spreading resistance genes^[47]^. In general, there is limited information available on the genetic basis of resistance in bifidobacteria. The genus is known to harbor resistance genes, such as *ermX*, *rpoB*, and *tetW*, on its chromosome, which confer resistance to erythromycin, rifamycin, and tetracycline, respectively^[22]^. Additionally, resistance genes like *cmX* and *tetQ* are found on plasmids and provide resistance to chloramphenicol and tetracycline^[22]^. The only resistance gene identified in this study was the *tetW* gene in *B. adolescentis* DX_pv1, conferring the strain with tetracycline resistance. The *tetW* gene is located on the chromosome and is generally considered a stable genetic element^[48]^. However, in some strains, the *tetW* gene is flanked by transposase target sequences or transposase-coding genes, suggesting it can be transferred under certain conditions. A low frequency of transfer has indeed been observed between *B. longum* and *B. adolescentis*^[48]^. While antimicrobial resistance is a major global healthcare concern, the presence of limited, non-transferable resistance in bacteriotherapeutic strains may offer certain advantages. For example, when these strains are administered alongside antimicrobials to which they are resistant, it may help ensure their survival while promoting host health. As discussed earlier, the bifidobacterial strains effectively prevent the adhesion of pathogens responsible for infections like urinary tract infections (e.g., *E. coli*)^[49]^. Moreover, our strains demonstrated resistance to antibiotics commonly used to treat these infections, such as ciprofloxacin and trimethoprim^[49]^. Therefore, these novel *Bifidobacterium* strains could serve a dual function: preventing infections linked to the human gut and supporting treatment efforts during ongoing infections. For instance, individuals traveling to regions with high exposure to ESBL-producing Enterobacteriaceae may benefit from these strains in infection prevention and treatment scenarios^[50]^.

There are some limitations that should be kept in mind when interpreting the results of this study. First, the limited number of pathogens examined narrows the scope of our findings. While we provided insights into the exclusion of certain pathogens by bifidobacterial strains, expanding the range of pathogens could provide a more comprehensive understanding of strains’ efficacy. Another limitation is that we did not explore whether bifidobacterial strains could compete with pathogens when present simultaneously or displace already adhered pathogens. Our study focused solely on bifidobacterial exclusion, meaning we did not address whether these strains could actively disrupt existing pathogen-mucus interactions or merely prevent initial adhesion. Future studies should investigate the ability of these strains to displace adhered pathogens, as this would provide critical insight into their potential therapeutic use in infections. Another major limitation relates to the use of RNA-Seq, which provided insights into gene expression during bifidobacterial exclusion of pathogens. While this approach was a strength of the study, as it has not been used extensively to explain how even poorly adherent bifidobacterial strains prevent pathogen adhesion, we did not directly link RNA-Seq findings to mucus degradation. This could be addressed in future studies by employing targeted knockouts or enzymatic assays to confirm the role of bifidobacterial enzymes in modifying the properties of mucus. Additionally, we did not explore whether metabolites produced by bifidobacterial strains might have inhibited pathogen adhesion by antimicrobial properties. Testing spent culture medium could clarify whether secreted metabolites contribute to exclusion, adding depth to understanding how these strains function in pathogen prevention. A further limitation is that we did not directly assess whether the bifidobacterial strains modify mucus composition through degradation, which could clarify their underlying mechanism of action. The antimicrobial susceptibility findings in the study also had some gaps. Although we assessed the resistance profiles of bifidobacterial strains, we did not fully elucidate the genetic mechanisms underlying much of the observed resistance. While we identified the *tetW* gene in *B. adolescentis* DX_pv1, other genetic bases for resistance observed *in vitro* were not identified. Additionally, although the antimicrobial panel used in this study was comprehensive, covering clinically relevant drugs, it did not include chloramphenicol, kanamycin, and streptomycin, which are recommended by EFSA for testing with probiotics^[51]^. We chose to focus on antimicrobials widely used in clinical practice in this initial phase, but future studies should include these drugs to meet broader testing standards and provide a complete resistance profile.

In summary, this study further assessed the therapeutic potential of promising bifidobacterial strains originating from fecal donors. Our results show that the strains are effective in inhibiting pathogen adhesion and we propose that efficient exclusion results from the degradation of intestinal mucus by specific hydrolases. Our strains do not show appreciable resistance to common antimicrobials, allowing their safe use in bacteriotherapeutic applications. The next logical step for studying the bifidobacterial donor strains would be a well-structured, double-blind, placebo-controlled trial that could help uncover their therapeutic potential. One critical aspect of such a study would be the delivery, with two main approaches standing out: encapsulated probiotics, which allow precise dosing and protect the bacteria during digestion, and functional foods, which provide an easy, everyday approach to probiotic intake. However, further studies are needed to optimize bacterial viability in these food matrices and assess their health benefits. Mucin degradation, a process crucial for intestinal mucus turnover, is a normal function of many gut bacteria, including bifidobacteria. While some bifidobacteria degrade mucins for carbon and energy, they also stimulate the ongoing production of mucins, helping maintain the mucosal barrier^[18]^. Incorporating this understanding into clinical studies could help clarify the role of bifidobacteria in supporting gut integrity, especially when conducting clinical trials with conditions plagued with impaired mucus function.

## DECLARATIONS

### Availability of data and materials

The whole-genome sequence data of the *Bifidobacterium* spp. strains used in the study were originally deposited in the European Nucleotide Archive (ENA) under the accession number PRJEB35833^[23]^. The RNA-Seq data for the strains were originally deposited to the National Center for Biotechnology Information (NCBI) Sequence Read Archive (SRA) under the accession number PRJNA930167^[24]^.

### Copyright

© The Author(s) 2024.
