# Supplementary material for: Pathogen exclusion from intestinal mucus and antimicrobial susceptibility of Bifidobacterium spp. strains from fecal donors
Source: Microbiome Res Rep. 2024 Nov 1;4(1):5. doi: 10.20517/mrr.2024.43 (PMC11977350; doi:10.20517/mrr.2024.43)
Supplement: Supplementary file 1 [file mrr-4-1-5-SupplementaryMaterials.pdf]

Supplementary Materials

Pathogen exclusion from intestinal mucus and antimicrobial susceptibility of *Bifidobacterium* spp. strains from fecal donors

Aki Ronkainen, Imran Khan, Reetta Satokari

Human Microbiome Research Program, Faculty of Medicine, University of Helsinki, Helsinki 00290, Finland.

Correspondence to: Reetta Satokari, Human Microbiome Research Program, Faculty of Medicine, University of Helsinki, Haartmaninkatu 3, Helsinki 00290, Finland.
E-mail: reetta.satokari@helsinki.fi

Supplementary Table 1. Differential glycoside hydrolase and glycosyl transferase expression between poorly adhering and adherent *B. longum* strains

Expression	Sequence name	Description	Enzyme Code	Reads
down in DX_pv18 DX_pv32	DX_32C_00006_xynB	glycoside hydrolase family 43 protein	3.2.1	-4.15
	DX_32C_00007_group_3034	glycoside hydrolase family 43 protein	3.2.1	-2.54
	DX_32C_00399_lnpA	1,3-beta-galactosyl-N-acetylhexosamine phosphorylase	2.4.1	-4.15
	DX_32C_00645_group_2865	FIVAR domain-containing protein	3.2.1	-4.77
	DX_32C_00802_treC	glycoside hydrolase family 13 protein	3	-3.02
	DX_32C_00803_group_2222	alpha-galactosidase	not given	-2.16
	DY_pv11_00399_group_1612	glycoside hydrolase family 43 protein	3.2.1	-681.37
	DY_pv11_00669_toxA	glycoside hydrolase	3.2.1	-1478.53
	DY_pv11_00788_group_1648	cellulase family glycosylhydrolase	3.2.1	-145.29
up in DX_pv18 DX_pv32	DX_32C_01967_bgaB_2	beta-galactosidase	3.2.1	20.35
	DX_32C_02183_group_1038	glycosyltransferase family 4 protein	2.4.1	2981.47
	DX_32C_02184_bshA	glycosyltransferase	2	1247.96
	DX_pv18_01905_group_1376	glycoside hydrolase	3.2.1	328.55

Transcriptome-based analysis was performed by comparing poorly adhering *B. longum* strains DX_pv18 and DX_pv32 with adherent *B. longum* strains DX_pv23 and DY_pv11.

Supplementary Table 2. Differential expression of glycoside hydrolase and glycosyl transferases among *B. longum* strains

Expression	Sequence name	Description	Enzyme Code	Reads
down in DX_pv18 DX_pv32 DY_pv11	DX_32C_00006_xynB	glycoside hydrolase family 43 protein	3.2.1	-4.28
	DX_32C_00062_group_144	glycoside hydrolase family 3 C-terminal domain-containing protein	3.2.1	-2.67
	DX_32C_00846_sacA	glycoside hydrolase family 32 protein	3.2.1	-3.69
	DX_32C_01427_beta-galIII	beta-galactosidase	3.2.1	-4.23
	DX_32C_01459_group_3044	bacterial Ig-like domain-containing protein	3.2.1	-2.64
up in DX_pv18 DX_pv32 DY_pv11	DX_32C_02183_group_1038	glycosyltransferase family 4 protein	2.4.1	1988.47
	DX_pv18_01031_group_440	family 43 glycosylhydrolase	3.2.1	1677.46
	DX_pv18_01032_group_1274	family 43 glycosylhydrolase	3.2.1	576.20
	DX_pv18_01033_group_93	Ig-like domain-containing protein	3.2.1	492.06
	DX_pv18_01085_mngB	alpha-mannosidase	3.2.1	829.95
	DX_pv18_01086_group_1275	alpha-mannosidase	3.2.1	489.88
	DX_pv18_01090_group_1279	glycosyl hydrolase	3	271.72
	DX_pv18_01091_group_1280	beta-glucosidase	3.2.1	85.49
	DX_pv18_01092_group_1281	Endo-beta-N-acetylglucosaminidase	3.2.1	258.22
	DX_pv18_01549_group_1346	metallophosphoesterase family protein	3	476.95
	DX_pv18_01629_group_456	carbon-nitrogen hydrolase family protein	3	142.68

Analysis was made by comparing the *B. longum* strains DX_pv18, DX_pv32, and DY_pv11 that showed high pathogen exclusion capacity with the *B. longum* DX_pv23 that was less effective in pathogen exclusion.
